# Recent Developments in Deciphering the Biological Role of Plant Complex *N*-Glycans

**DOI:** 10.3389/fpls.2022.897549

**Published:** 2022-04-25

**Authors:** Richard Strasser

**Affiliations:** Department of Applied Genetics and Cell Biology, Institute of Plant Biotechnology and Cell Biology, University of Natural Resources and Life Sciences, Vienna, Austria

**Keywords:** glycan, glycoprotein, glycosylation, posttranslational modification, secretory pathway

## Abstract

Asparagine (*N*)-linked protein glycosylation is a ubiquitous co- and posttranslational modification which has a huge impact on the biogenesis and function of proteins and consequently on the development, growth, and physiology of organisms. In mammals, *N*-glycan processing carried out by Golgi-resident glycosidases and glycosyltransferases creates a number of structurally diverse *N*-glycans with specific roles in many different biological processes. In plants, complex *N*-glycan modifications like the attachment of β1,2-xylose, core α1,3-fucose, or the Lewis A-type structures are evolutionary highly conserved, but their biological function is poorly known. Here, I highlight recent developments that contribute to a better understanding of these conserved glycoprotein modifications and discuss future directions to move the field forward.

## Introduction

*N*-glycosylation of proteins is initiated in the lumen of the endoplasmic reticulum (ER) by the oligosaccharyltransferase (OST) complex which transfers a preassembled oligosaccharide to an asparagine residue within the Asn-X-Ser/Thr consensus sequence of a nascent polypeptide. While the total number of proteins in the plant *N*-linked glycoproteome is unknown, every protein that has the consensus *N*-glycosylation site in its sequence and is targeted to the secretory pathway is a potential substrate for *N*-glycosylation and *N*-glycan dependent folding. The transferred oligosaccharide can directly influence polypeptide folding by stabilizing protein conformations. In addition to the direct effect of the attached oligosaccharide, specific *N*-glycans are recognized as signals by lectins which assist in protein folding, retain folding intermediates in the ER, or trigger ER-associated degradation (ERAD) if proper folding cannot be achieved (Strasser, 2018; Zhang et al., 2021).

**Figure 1:**
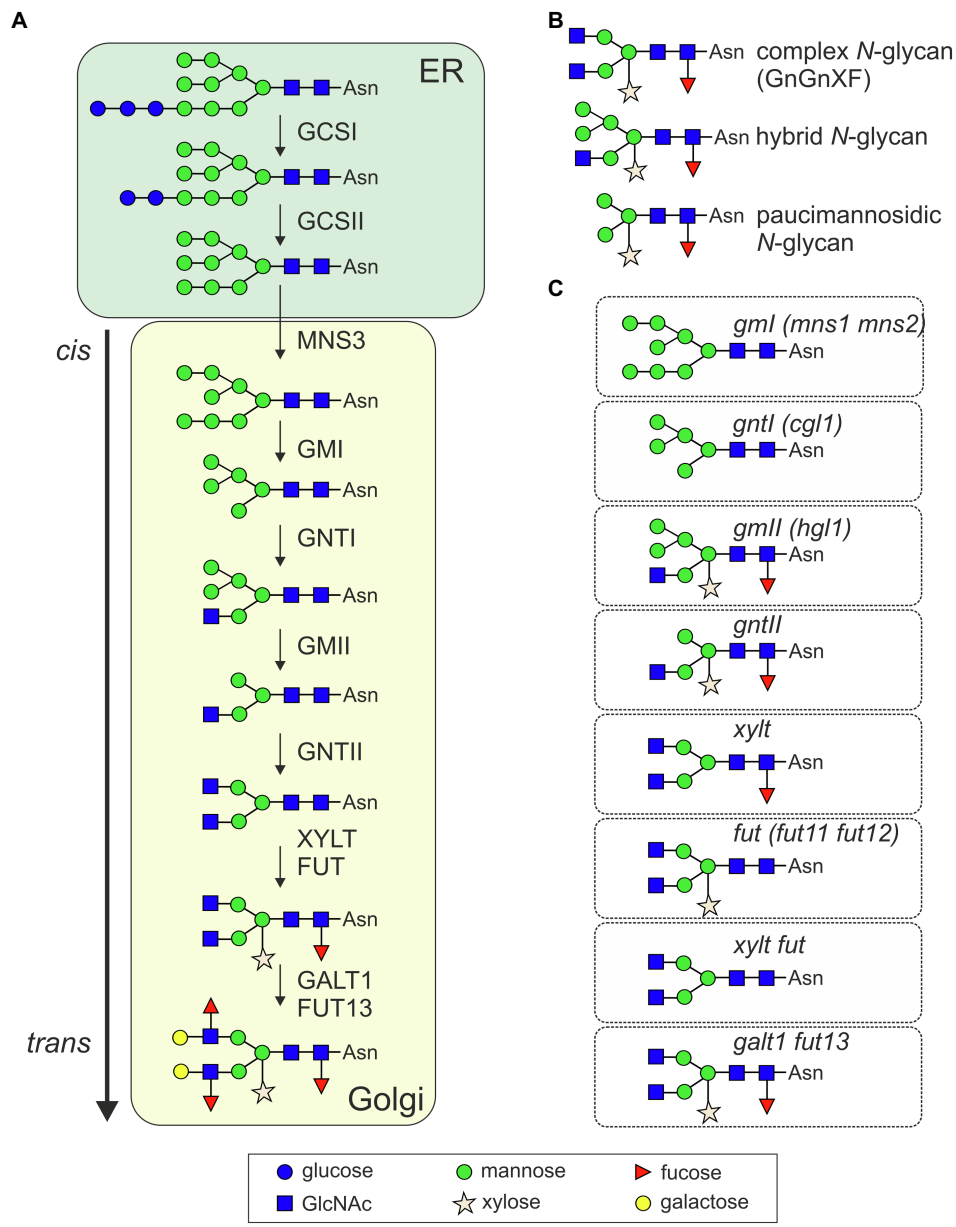
**(A)** Illustration of the processing pathway for the formation of complex *N*-glycans in plants. GCSI, α-glucosidases I (GCSI); GCSII, α-glucosidases II; MNS3, ER α-mannosidase; GMI, Golgi α-mannosidase I (two forms termed MNS1 and MNS2 with redundant function are present in *Arabidopsis thaliana*); GNTI, β1,2-*N*-acetylglucosaminyltransferase I; GMII, Golgi α-mannosidase II; GNTII, β1,2-*N*-acetylglucosaminyltransferase II; XYLT, β1,2-xylosyltransferase; FUT, core α1,3-fucosyltransferases (two forms termed FUT11 and FUT12 with redundant function are present in *A. thaliana*); GALT1, Lewis type β1,3-galactosyltransferase; and FUT13, α1,4-fucosyltransferase. Not shown: the removal of terminal GlcNAc residues by β-hexosaminidases, which generates paucimannosidic *N*-glycans in post-Golgi compartments, at the plasma membrane or in the extracellular space. **(B)** Illustration of typical complex, hybrid, and paucimannosidic *N*-glycans. **(C)** Illustration of the predominate *N*-glycan structure of the indicated knockout mutants. Alternative names of the mutants are given in brackets. Symbols are used according to the suggestions from the Consortium for Functional Glycomics (http://www.functionalglycomics.org/).

Initial trimming by ER-resident α-glucosidases (GCSI and GCSII, Figure 1A) generates a monoglucosylated *N*-glycan that allows the transient interaction with the lectins calnexin or calreticulin and entry into an ER-quality control cycle. Further trimming of mannose residues is carried out by ER- (MNS3) and Golgi-α-mannosidases (GMI; Liebminger et al., 2009; Kajiura et al., 2010). The resulting Man_5_GlcNAc_2_ *N*-glycan is used as acceptor substrate by the *cis*/*medial*-Golgi-resident *N*-acetylglucosaminyltransferase I (GNTI). GNTI transfers a single *N*-acetylglucosamine (GlcNAc) residue and initiates the formation of characteristic complex *N*-glycans carrying β1,2-linked xylose and an α1,3-fucose attached to the innermost GlcNAc, respectively (GnGnXF structures, Figure 1B). While the core complex *N*-glycan is identical in mammals and plants, β1,2-xylose and core α1,3-fucose modifications are not found on mammalian glycoproteins (Strasser, 2016). Their biosynthesis is carried out by β1,2-xylosyltransferase (XYLT) and core α1,3-fucosyltransferase (FUT; Strasser et al., 2004). While GnGnXF is the dominant structure on many glycoproteins (Zeng et al., 2018), on a rather small number of glycoproteins, the GnGnXF *N*-glycan is further modified by β1,3-galactosyltransferase (GALT1) and α1,4-fucosyltransferase (FUT13) to generate Lewis A-type structures (Strasser et al., 2007a; Beihammer et al., 2021). In the vacuole, at the plasma membrane or in the apoplast, different β-hexosaminidases can cleave off terminal GlcNAc residues from exposed complex *N*-glycans resulting in the formation of truncated or paucimannosidic *N*-glycans (Strasser et al., 2007b; Alvisi et al., 2021). The biosynthetic pathway and involved enzymes are well characterized and have been reviewed in detail recently (Strasser et al., 2021). Notably, the whole machinery for complex *N*-glycan formation appears conserved in vascular plants and in mosses like *Physcomitrella patens* (Koprivova et al., 2003; Viëtor et al., 2003; Strasser, 2016; Stenitzer et al., 2022). Complex GnGnXF and Lewis A-type structures are ubiquitously found in plants (Fitchette-Lainé et al., 1997; Wilson et al., 2001; Beihammer et al., 2021) which point to a selective pressure and a functional advantage to maintain these *N*-glycans. Despite this conservation, the knowledge about the physiological role of complex *N*-glycan modifications is still limited. However, in the last decade, considerable progress has been made and different processes have been revealed where complex *N*-glycans play an important role in plant physiology, development, and under various stress conditions. This has been spurred by in-depth analysis of Arabidopsis mutants and gene knockouts generated by CRISPR/Cas9 genome editing or other technologies in different plant species.

## The Achievements in the Past Decade: Deciphering the Biological Role of Complex *N*-Glycans by Phenotypic Characterization of Gene Knockouts

Our knowledge about the role of complex *N*-glycans stems mainly from the analysis of mutants deficient in *N*-glycan processing steps. The Arabidopsis *complex glycan1* (*cgl1* or *gntI*) mutant that lacks GNTI activity and thus displays Man_5_GlcNAc_2_ instead of complex *N*-glycans was first described almost three decades ago (von Schaewen et al., 1993; Figure 1C). In this pioneering study on the role of complex *N*-glycans in plants, no obvious growth or developmental phenotype was reported for *cgl1* plants grown under controlled growth conditions. This lack of a severe phenotype in Arabidopsis *cgl1* was a surprise because the consequence of GNTI-deficiency in mammals was embryonic lethality (Ioffe and Stanley, 1994; Metzler et al., 1994). Later it was, however, shown that Arabidopsis *cgl1* displayed reduced root growth when subjected to osmotic stress or high salt concentrations (Kang et al., 2008) and a recent study observed that *cgl1* has reduced photosynthetic efficiency (Jiao et al., 2020). This link to photosynthesis suggests that glycoproteins carrying complex *N*-glycans are transported from the Golgi apparatus or a post-Golgi compartment to chloroplasts where they fulfill important functions. Of note, a detailed analysis of the root system in Arabidopsis seedlings showed that root hairs are significantly longer in *cgl1* plants which for the first time revealed a developmental phenotype in Arabidopsis plants with abrogated complex *N*-glycan formation (Frank et al., 2021). The *cgl1* roots appeared generally more responsive to synthetic phytohormones suggesting that complex *N*-glycans on one or several glycoproteins are critical for phytohormone homeostasis.

A knockdown of *GnTI* expression in *Nicotiana benthamiana* resulted in a decrease in the amounts of complex type *N*-glycans from 90% to less than 10% without any growth or reproduction defects (Limkul et al., 2016). This is consistent with previous data showing that *N. benthamiana*, which is frequently used as a production platform for protein-based biopharmaceuticals, tolerates the virtual absence of GnGnXF *N*-glycans very well (Strasser et al., 2008). While a *GnTI* knockdown in tomato resulted in abnormal fruit ripening (Kaulfürst-Soboll et al., 2021), an *Oryza sativa* GNTI-deficient line that displayed a similar *N*-glycan profile as Arabidopsis *cgl1* showed a severe growth phenotype with arrested seedling development and lethality before reaching the reproductive stage (Fanata et al., 2013). In line with this finding, *GnTI* gene disruption in the legume *Lotus japonicus* caused a severe growth defect with lethality before reaching the flowering stage (Pedersen et al., 2017). Taken together, our knowledge on the role of GNTI in Arabidopsis and other plants has enormously increased in the last decade.

Upon transfer of the single GlcNAc by GNTI, two mannose residues are removed by Golgi-α-mannosidase II (GMII). In contrast to GNTI, GMII deficiency does not block further processing completely and results in hybrid *N*-glycans that are still modified with β1,2-xylose and core α1,3-fucose (Strasser et al., 2006; Kaulfürst-Soboll et al., 2011). Tomatoes with reduced *GMII* expression produced fewer and enlarged seeds (Kaulfürst-Soboll et al., 2021) and Arabidopsis *gmII* plants displayed similar differences in root hair density and length as Arabidopsis *cgl1*/*gntI* (Frank et al., 2021). The finding in Arabidopsis suggests that terminal modifications (presence of mannose residues or absence of GlcNAc residues on both antennae or blocked terminal elongations) rather than core glycan modifications (attachment of xylose or fucose) are important for the function in root hair elongation (Figure 1C). Consistent with this, *galt1 fut13* double mutants lacking *N*-glycan modifications that form the Lewis A structure are partially impaired in root hair elongation (Frank et al., 2021) indicating a specific role for these terminal complex *N*-glycan modifications.

After the removal of the two terminal mannose residues by GMII, the free α1,6-mannose of the core glycan is used by *N*-acetylglucosaminyltransferase II (GNTII) for the attachment of a single GlcNAc residue (Strasser et al., 1999). Consequently, *GnTII* loss-of-function in Arabidopsis prevents the formation of GnGnXF glycans and *gntII* mutants displayed numerous phenotypes including slightly early flowering, accelerated dark-induced leaf senescence and altered responses to NaCl, the *N*-glycosylation inhibitor tunicamycin, a synthetic cytokinin, and a polar auxin transport inhibitor (Yoo et al., 2021). These pleiotropic effects suggest that several glycoproteins involved in different processes are affected in the *gntII* mutant. Consistent with the impaired auxin transport, the abundance of the auxin transporter PIN2 fused to GFP and its subcellular localization were altered in *gntII*. However, PIN2 is likely not *N*-glycosylated because all potential *N*-glycosylation sites are in the hydrophilic loop that faces the cytosol. Therefore, it is more likely that other glycoproteins involved in auxin signaling or transport are directly affected by incomplete processing of the α1,6-mannose on the complex *N*-glycan.

No phenotype has so far been described for the Arabidopsis FUT loss-of-function mutant lacking core α1,3-fucose residues (Strasser et al., 2004). In line with the crucial role of complex *N*-glycans in other plant species, growth defects, reduced seed number, impaired pollen viability, and morphology were observed in rice and *L. japonicus fut* lines (Harmoko et al., 2016; Pedersen et al., 2017; Sim et al., 2018). By contrast, genome-edited *Nicotiana benthamiana* completely lacking β1,2-xylose and core α1,3-fucose did not display any growth abnormalities or reproduction defects (Jansing et al., 2019). Overall, the function of the β1,2-xylose residue is poorly understood, and phenotypes have only been described for a rice XYLT-deficient mutant. Rice *xylt* plants are impaired in their growth under adverse environmental conditions including extreme temperatures, drought, or salt stress (Takano et al., 2015). Rice callus lacking XYLT and FUT activities grew normal, but plants could not be regenerated from *xylt fut* rice callus which is likely caused by the altered phytohormone responsiveness (Jung et al., 2021). Taken together, these findings indicate that complex *N*-glycans are essential in many plant species for growth, reproduction, phytohormone homeostasis, and for the response to different stresses (Table 1). In Arabidopsis, complex *N*-glycans play an important role for root hair elongation in addition to the role in abiotic stress tolerance. Like pollen, root hairs are formed through tip growths and secrete numerous cell wall components which could be impaired in the *N*-glycan processing mutants. In line with described phenotypes for pollen, FUT13 which catalyzes the final *N*-glycan processing step in the Golgi (Figure 1) appears highly regulated during pollen development (Joly et al., 2002). Transcription of glycosidases or glycosyltransferases involved in *N*-glycan processing provides another level for regulating the abundance of certain *N*-glycan structures. While glycoenzyme-specific transcription factors have not been identified in plants yet, the transcriptional regulation of *GALT1* and *FUT13* likely contributes to the abundance of Lewis A structures in different Arabidopsis organs (Strasser et al., 2007a; Stefanowicz et al., 2016). Furthermore, β-hexosaminidases or other degrading glycosidases that act on complex *N*-glycans may have a physiological role that is largely unknown (Strasser et al., 2007b; Kato et al., 2018).

**Table 1:** Overview of phenotypes in vascular plants with aberrant *N*-glycans.

Species	Gene^1^	Technology^2^	Phenotype^3^	Reference
*Arabidopsis thaliana*	*GMI*	T-DNA	Altered root morphology No obvious phenotype Increased salt sensitivity Increased sensitivity to *Pseudomonas syringae*	Liebminger et al., 2009 Kajiura et al., 2010 Liu et al., 2018 Jia et al., 2020
	*GnTI*	EMS/T-DNA	No obvious phenotype Increased salt sensitivity Decreased photosynthetic efficiency Altered auxin responsiveness Altered root hair development	^58,22^ Jiao et al., 2020 Frank et al., 2021
	*GMII*	T-DNA	No obvious phenotype Increased salt sensitivity Altered auxin responsiveness Altered root hair development	Strasser et al., 2006 Kang et al., 2008 Kaulfürst-Soboll et al., 2011 Frank et al., 2021
	*GnTII*	T-DNA	Altered growth under stress Altered phytohormone response	Yoo et al., 2021
	*Xylt*	T-DNA	No obvious phenotype	Strasser et al., 2004 Kang et al., 2008
	*Fut*	T-DNA	No obvious phenotype	Strasser et al., 2004 Kang et al., 2008
	*Xylt + Fut* *Galt1 + Fut13*	T-DNA T-DNA	No obvious phenotype Increased salt sensitivity No obvious phenotype Altered root hair development	Strasser et al., 2004 Kang et al., 2008 Strasser et al., 2007a Frank et al., 2021
*Oryza sativa*	*GnTI*	T-DNA	Reduced growth, altered seed set	Fanata et al., 2013
	*Xylt*	Gamma-ray	Plant growth affected under various stresses, affected seed germination	Takano et al., 2015
	*Fut*	T-DNA	Pollen viability affected	Sim et al., 2018
	*Fut*	T-DNA	Developmental abnormalities Increased sensitivity to *Magnaporthe oryzae*	Harmoko et al., 2016
	*Xylt + Fut*	CRISPR/Cas9	No phenotype in callus	Jung et al., 2021
*Lotus japonicus*	*GMI*	LORE1 retrotransposon	Reduced growth Reduced seed number	Pedersen et al., 2017
	*GnTI*	LORE1 retrotransposon	Severe growth defect, lethality	Pedersen et al., 2017
	*Fut*	LORE1 retrotransposon	Reduced growth Reduced seed number	Pedersen et al., 2017
*Nicotiana*	*GnTI*	RNAi	No obvious phenotype	Limkul et al., 2016
*benthamiana*	*Xylt*	CRISPR/Cas9	No obvious phenotype	Jansing et al., 2019
	*Fut*	CRISPR/Cas9	No obvious phenotype	Jansing et al., 2019
	*Xylt + Fut*	CRISPR/Cas9	No obvious phenotype	Jansing et al., 2019
	*Xylt + Fut*	RNAi	No obvious phenotype	Strasser et al., 2008
*Nicotiana tabacum*	*GnTI*	antisense	No obvious phenotype	Wenderoth and von Schaewen, 2000
*tobacco BY-2*	*GnTI*	CRISPR/Cas9	No obvious phenotype	Herman et al., 2021
*cells*	*Xylt + Fut*	CRISPR/Cas9	No obvious phenotype	Hanania et al., 2017
	*Xylt + Fut*	CRISPR/Cas9	No obvious phenotype	Mercx et al., 2017
*Solanum*	*GnTI*	RNAi	Abnormal fruit ripening	Kaulfürst-Soboll et al., 2021
*lycopersicum*	*GMII*	RNAi	Fewer, enlarged seeds	Kaulfürst-Soboll et al., 2021
*Solanum tuberosum*	*GnTI*	antisense	No obvious phenotype	Wenderoth and von Schaewen, 2000

1 *N*-glycan processing defects of Golgi located enzymes are listed.

2 This indicates the technology used to generate the mutants.

3 For mutants with various phenotypes, only some characteristic phenotypes are shown.

## The challenge for the Next 10 years: Understanding the Role of Distinct *N*-Glycan Modifications on Individual Glycoproteins to Obtain Mechanistic Insights

For almost all the identified biological processes where complex *N*-glycans are involved, an insight into the underlying mechanisms and molecular function of a distinct complex *N*-glycan linked to an individual protein is missing. The disadvantage of characterization of *N*-glycan processing mutants is their pleiotropic effect on numerous glycoproteins and an inherent difficulty to identify individual *N*-glycans on a distinct glycoprotein that are involved in a specific process. In a recent glycoproteome study (Liu et al., 2021), differentially abundant proteins were identified in Arabidopsis wild type, *cgl1* and the *gmI* mutant when grown under elevated salt conditions. Among the proteins increased under salt stress were proteins involved in glycoprotein biosynthesis, stress response, signal transduction, and oxidation–reduction processes. Two peroxidases (PRX32 and PRX34) were, for example, differentially abundant in the mutants under salt stress. The two peroxidases have several potential *N*-glycosylation sites in their amino acid sequence, were *N*-glycosylated, and seedlings of the peroxidase double knockout mutant were salt-sensitive (Liu et al., 2021). While such studies provide a starting point for the characterization of *N*-glycan modifications on individual proteins, there is no direct evidence that the *N*-glycosylation and distinct *N*-glycan modifications are indeed relevant for the function of the two peroxidases. Because of impaired complex *N*-glycan processing, many different processes are affected leading to massive changes in the transcriptome and proteome that are indirect due to alterations of key signaling pathways (Sim et al., 2018). In mammalian cells, there is evidence that Golgi-mediated complex *N*-glycan modifications like core α1,6-fucosylation regulate the *N*-glycosylation efficiency on different glycoproteins (Huang et al., 2021). The regulation of the upstream *N*-glycosylation process catalyzed by the ER-resident OST complex by downstream *N-*glycan modifications could be mediated by Golgi feedback events as part of poorly understood Golgi-quality control mechanisms (Sun and Brodsky, 2019). This further complicates the interpretation of quantitative glycoproteomics data obtained from *N*-glycan processing mutants. Moreover, underglycosylated proteins are more instable, prone to aggregation and therefore less efficiently enriched by commonly used lectin-based affinity purification approaches. Lectins typically show a preference for a certain type of *N*-glycan causing a bias when samples with different *N*-glycan compositions are analyzed. Collectively, this makes the comparison of the *N*-glycoproteome abundance from different mutants impossible. Even when all these difficulties can be overcome, a confirmed differentially abundant glycoprotein with altered *N*-glycans in a mutant background might not be involved in the process and a comprehensive characterization of the glycoprotein and its fate in the mutant background are required to unravel the underlying mechanisms. Approaches to understand the role of complex *N*-glycans on individual proteins include the analysis of the molecular function (e.g., enzymatic activity), protein stability, cellular interaction partners, and the subcellular localization when the *N*-glycan composition is altered on the protein. As mentioned before, the difficulty is to distinguish between specific and pleiotropic effects in the mutant background. Despite some tremendous progress in glycoengineering in plants (Schoberer and Strasser, 2018), controlled site-specific modification of *N*-glycans (e.g., to furnish one *N*-glycan with Lewis A-type structures while another *N*-glycan attached to a different position on the same protein stays GnGnXF) is currently not possible *in vivo*. Therefore, multiple tedious approaches are required with the generation of mutant variants that lack individual *N*-glycosylation sites and careful examination of the protein fate and function.

An in-depth analysis of *N*-glycan function has been done for KORRIGAN 1 (KOR1), a β1,4-endoglucanase involved in cellulose biosynthesis (Lane et al., 2001; Liebminger et al., 2013; Rips et al., 2014; Nagashima et al., 2020). KOR1 is a membrane-anchored glycoprotein with eight *N*-glycosylation sites in its extracellular domain. *N*-glycan analysis of a recombinant variant as well as the analysis of *N*-glycosylation site mutants expressed in plants confirmed that all sites are *N*-glycosylated (Liebminger et al., 2013; Rips et al., 2014). KOR1 is glycosylated with oligomannosidic *N*-glycans as well as with Golgi-processed complex ones. While *N*-glycosylation is essential for the enzymatic function, analysis of purified recombinant KOR1 carrying different *N*-glycan compositions showed that *N*-glycan processing in the Golgi is not important for KOR1 enzymatic activity (Mølhøj et al., 2001; Liebminger et al., 2013). Of note, genetic interaction analysis between a KOR1 partial loss-of-function allele (*rsw2-1*) and *N*-glycan processing mutants like *cgl1* revealed strong synergistic effects on the plant growth (Kang et al., 2008). Moreover, a non-glycosylated KOR1 variant could partially complement the root growth phenotype of a KOR1-deficient mutant (Rips et al., 2014). The data from the enzymatic activity assays and complementation of the *rsw2-1* mutant strongly suggest that other glycoproteins are affected which require complex *N*-glycans for their function and thus contribute to the phenotype when KOR1 is compromised (Liebminger et al., 2013; Rips et al., 2014; Nagashima et al., 2020). Biochemical analysis indicated that KOR1 protein abundance is affected under salt stress when mannose-trimming is blocked in the Arabidopsis *gmI* mutant suggesting the involvement of a yet to be discovered mechanism that regulates glycoprotein stability or trafficking under different environmental conditions (Liu et al., 2018). Taken together, these findings highlight the complexity in the analysis of *N*-glycan function on individual proteins and the difficulty to separate effects from oligomannosidic *N*-glycans in the ER (e.g., protein folding, quality control, and degradation) and processed *N*-glycans in the Golgi (e.g., conformational changes and protein–protein interactions).

To overcome current hurdles and move the field forward, more efforts should be made to purify individual glycoproteins from different plant organs, cell-types, or different growth conditions and perform a comprehensive analysis of their *N*-glycosylation status including the number of *N*-glycans, the degree of site occupancy, and the *N*-glycan composition. Using such approaches, it may be possible to reveal potential changes in *N*-glycan structures on an individual glycoprotein that point toward a specific function during development or under stress conditions. While there are indications that *N*-glycans vary under different growth conditions or developmental stages (Elbers et al., 2001; Horiuchi et al., 2016; Kaulfürst-Soboll et al., 2021), the underlying cause of the differences is less clear. Changes may be attributed to variations in protein abundance, expression of glycoproteins, *N*-glycosylation efficiency, or altered *N*-glycan structures. In addition to ER-quality control, there are data suggesting a role of lectin-glycoprotein interactions in the Golgi, *trans*-Golgi network (TGN) or another post-Golgi compartment that may provide another layer of quality control or regulation of transport to specific organelles (Rips et al., 2014; Liu et al., 2018; Veit et al., 2018; Nagashima et al., 2020). In the future, we will gain more insights into these processes involving complex *N*-glycans.

The conserved nature of many complex *N*-glycan modifications could be the result of a selection pressure mediated by pathogens (Gagneux and Varki, 1999). While the impact of *N*-glycosylation and glycan-mediated folding on pattern recognition receptors is well known (Nekrasov et al., 2009; Saijo et al., 2009; Häweker et al., 2010), the role of complex *N*-glycans under biotic stress conditions is still poorly understood. The Arabidopsis *gmI* mutant is more susceptible to *Pseudomonas syringae* (Jia et al., 2020) and rice *fut* plants are more susceptible to *Magnaporthe oryzae* (Harmoko et al., 2016) providing hints that complex *N*-glycans have crucial roles in plant immunity during pathogen infection. In the next decade, we will likely uncover many more examples of interactions of plants with symbiotic or pathogenic organisms that depend on specific *N*-glycan modifications and interacting lectins. In conclusion, numerous biological processes with functional roles of complex *N*-glycans are known now. These discoveries lay the foundation to examine the role of complex *N*-glycans on individual proteins and decipher the underlying mechanisms in the fascinating world of plant protein glycosylation.

## Author Contributions

RS wrote and edited the manuscript. The author confirms being the sole contributor of this work and has approved it for publication.

## Funding

This work was supported by the Austrian Science Fund (FWF) Project P31920-B32.

## Conflict of Interest

The author declares that the research was conducted in the absence of any commercial or financial relationships that could be construed as a potential conflict of interest.

## Publisher’s Note

All claims expressed in this article are solely those of the authors and do not necessarily represent those of their affiliated organizations, or those of the publisher, the editors and the reviewers. Any product that may be evaluated in this article, or claim that may be made by its manufacturer, is not guaranteed or endorsed by the publisher.

## References

[1] Alvisi N. Van Noort K. Dwiani S. Geschiere N. Sukarta O. Varossieau K.. (2021). β-Hexosaminidases along the secretory pathway of *Nicotiana benthamiana* have distinct specificities toward engineered helminth N-glycans on recombinant glycoproteins. Front. Plant Sci. 12:638454. doi: 10.3389/fpls.2021.638454, PMID: 33815445 PMC8010188

[2] Beihammer G. Maresch D. Altmann F. Van Damme E. J. M. Strasser R. (2021). Lewis A glycans are present on proteins involved in cell wall biosynthesis and appear evolutionarily conserved among natural. Front. Plant Sci. 12:630891. doi: 10.3389/fpls.2021.630891, PMID: 33777069 PMC7991798

[3] Elbers I. J. Stoopen G. M. Bakker H. Stevens L. H. Bardor M. Molthoff J. W.. (2001). Influence of growth conditions and developmental stage on N-glycan heterogeneity of transgenic immunoglobulin G and endogenous proteins in tobacco leaves. Plant Physiol. 126, 1314–1322. doi: 10.1104/pp.126.3.1314, PMID: 11457982 PMC116488

[4] Fanata W. I. Lee K. H. Son B. H. Yoo J. Y. Harmoko R. Ko K. S.. (2013). N-glycan maturation is crucial for cytokinin-mediated development and cellulose synthesis in *Oryza sativa*. Plant J. 73, 966–979. doi: 10.1111/tpj.12087, PMID: 23199012

[5] Fitchette-Lainé A. Gomord V. Cabanes M. Michalski J. Saint Macary M. Foucher B.. (1997). N-glycans harboring the Lewis a epitope are expressed at the surface of plant cells. Plant J. 12, 1411–1417. doi: 10.1046/j.1365-313x.1997.12061411.x, PMID: 9450345

[6] Frank M. Kaulfürst-Soboll H. Fischer K. Von Schaewen A. (2021). Complex-type N-glycans influence the root hair landscape of Arabidopsis seedlings by altering the auxin output. Front. Plant Sci. 12:635714. doi: 10.3389/fpls.2021.635714, PMID: 33679849 PMC7930818

[7] Gagneux P. Varki A. (1999). Evolutionary considerations in relating oligosaccharide diversity to biological function. Glycobiology 9, 747–755. doi: 10.1093/glycob/9.8.747, PMID: 10406840

[9] Hanania U. Ariel T. Tekoah Y. Fux L. Sheva M. Gubbay Y.. (2017). Establishment of a tobacco BY2 cell line devoid of plant-specific xylose and fucose as a platform for the production of biotherapeutic proteins. Plant Biotechnol. J. 15, 1120–1129. doi: 10.1111/pbi.12702, PMID: 28160363 PMC5552476

[10] Harmoko R. Yoo J. Y. Ko K. S. Ramasamy N. K. Hwang B. Y. Lee E. J.. (2016). N-glycan containing a core α1,3-fucose residue is required for basipetal auxin transport and gravitropic response in rice (*Oryza sativa*). New Phytol. 212, 108–122. doi: 10.1111/nph.14031, PMID: 27241276

[11] Häweker H. Rips S. Koiwa H. Salomon S. Saijo Y. Chinchilla D.. (2010). Pattern recognition receptors require N-glycosylation to mediate plant immunity. J. Biol. Chem. 285, 4629–4636. doi: 10.1074/jbc.M109.063073, PMID: 20007973 PMC2836068

[12] Herman X. Far J. Courtoy A. Bouhon L. Quinton L. De Pauw E.. (2021). Inactivation of N-acetylglucosaminyltransferase I and α1,3-fucosyltransferase genes in *Nicotiana tabacum* BY-2 cells results in glycoproteins with highly homogeneous high-mannose N-glycans. Front. Plant Sci. 12:634023. doi: 10.3389/fpls.2021.634023, PMID: 33584780 PMC7873608

[13] Horiuchi R. Hirotsu N. Miyanishi N. (2016). N-glycan transition of the early developmental stage in *Oryza sativa*. Biochem. Biophys. Res. Commun. 477, 426–432. doi: 10.1016/j.bbrc.2016.06.082, PMID: 27320861

[14] Huang Y. Zhang H. L. Li Z. L. Du T. Chen Y. H. Wang Y.. (2021). FUT8-mediated aberrant N-glycosylation of B7H3 suppresses the immune response in triple-negative breast cancer. Nat. Commun. 12:2672. doi: 10.1038/s41467-021-22618-x, PMID: 33976130 PMC8113546

[15] Ioffe E. Stanley P. (1994). Mice lacking N-acetylglucosaminyltransferase I activity die at mid-gestation, revealing an essential role for complex or hybrid N-linked carbohydrates. Proc. Natl. Acad. Sci. U. S. A. 91, 728–732. doi: 10.1073/pnas.91.2.728, PMID: 8290590 PMC43022

[16] Jansing J. Sack M. Augustine S. M. Fischer R. Bortesi L. (2019). CRISPR/Cas9-mediated knockout of six glycosyltransferase genes in *Nicotiana benthamiana* for the production of recombinant proteins lacking β-1,2-xylose and core α-1,3-fucose. Plant Biotechnol. J. 17, 350–361. doi: 10.1111/pbi.12981, PMID: 29969180 PMC6335070

[17] Jia X. Zeng H. Bose S. K. Wang W. Yin H. (2020). Chitosan oligosaccharide induces resistance to Pst DC3000 in Arabidopsis via a non-canonical N-glycosylation regulation pattern. Carbohydr. Polym. 250:116939. doi: 10.1016/j.carbpol.2020.116939, PMID: 33049851

[18] Jiao Q. S. Niu G. T. Wang F. F. Dong J. Y. Chen T. S. Zhou C. F.. (2020). N-glycosylation regulates photosynthetic efficiency of *Arabidopsis thaliana*. Photosynthetica 58, 72–79. doi: 10.32615/ps.2019.153

[19] Joly C. Léonard R. Maftah A. Riou-Khamlichi C. (2002). alpha4-fucosyltransferase is regulated during flower development: increases in activity are targeted to pollen maturation and pollen tube elongation. J. Exp. Bot. 53, 1429–1436. doi: 10.1093/jexbot/53.373.1429, PMID: 12021290

[20] Jung J. W. Shin J. H. Lee W. K. Begum H. Min C. H. Jang M. H.. (2021). Inactivation of the β (1,2)-xylosyltransferase and the α (1,3)-fucosyltransferase gene in rice (*Oryza sativa*) by multiplex CRISPR/Cas9 strategy. Plant Cell Rep. 40, 1025–1035. doi: 10.1007/s00299-021-02667-8, PMID: 33547931

[21] Kajiura H. Koiwa H. Nakazawa Y. Okazawa A. Kobayashi A. Seki T.. (2010). Two *Arabidopsis thaliana* Golgi alpha-mannosidase I enzymes are responsible for plant N-glycan maturation. Glycobiology 20, 235–247. doi: 10.1093/glycob/cwp170, PMID: 19914916

[22] Kang J. Frank J. Kang C. Kajiura H. Vikram M. Ueda A.. (2008). Salt tolerance of *Arabidopsis thaliana* requires maturation of N-glycosylated proteins in the Golgi apparatus. Proc. Natl. Acad. Sci. U. S. A. 105, 5933–5938. doi: 10.1073/pnas.0800237105, PMID: 18408158 PMC2311335

[23] Kato S. Hayashi M. Kitagawa M. Kajiura H. Maeda M. Kimura Y.. (2018). Degradation pathway of plant complex-type N-glycans: identification and characterization of a key α1,3-fucosidase from glycoside hydrolase family 29. Biochem. J. 475, 305–317. doi: 10.1042/BCJ20170106, PMID: 29212795

[24] Kaulfürst-Soboll H. Mertens-Beer M. Brehler R. Albert M. von Schaewen A. (2021). Complex N-glycans are important for normal fruit ripening and seed development in tomato. Front. Plant Sci. 12:635962. doi: 10.3389/fpls.2021.635962, PMID: 33767719 PMC7985349

[25] Kaulfürst-Soboll H. Rips S. Koiwa H. Kajiura H. Fujiyama K. von Schaewen A. (2011). Reduced immunogenicity of Arabidopsis *hgl1* mutant N-glycans caused by altered accessibility of xylose and core fucose epitopes. J. Biol. Chem. 286, 22955–22964. doi: 10.1074/jbc.M110.196097, PMID: 21478158 PMC3123063

[26] Koprivova A. Altmann F. Gorr G. Kopriva S. Reski R. Decker E. (2003). N-glycosylation in the moss *Physcomitrella patens* is organized similarly to that in higher plants. Plant Biol. 5, 582–591. doi: 10.1055/s-2003-44721

[27] Lane D. Wiedemeier A. Peng L. Höfte H. Vernhettes S. Desprez T.. (2001). Temperature-sensitive alleles of RSW2 link the KORRIGAN endo-1,4-beta-glucanase to cellulose synthesis and cytokinesis in Arabidopsis. Plant Physiol. 126, 278–288. doi: 10.1104/pp.126.1.278, PMID: 11351091 PMC102302

[28] Liebminger E. Grass J. Altmann F. Mach L. Strasser R. (2013). Characterizing the link between glycosylation state and enzymatic activity of the endo-β1,4-glucanase KORRIGAN1 from *Arabidopsis thaliana*. J. Biol. Chem. 288, 22270–22280. doi: 10.1074/jbc.M113.475558, PMID: 23782689 PMC3829318

[29] Liebminger E. Hüttner S. Vavra U. Fischl R. Schoberer J. Grass J.. (2009). Class I alpha-mannosidases are required for N-glycan processing and root development in *Arabidopsis thaliana*. Plant Cell 21, 3850–3867. doi: 10.1105/tpc.109.072363, PMID: 20023195 PMC2814498

[30] Limkul J. Iizuka S. Sato Y. Misaki R. Ohashi T. Fujiyama K. (2016). The production of human glucocerebrosidase in glyco-engineered *Nicotiana benthamiana* plants. Plant Biotechnol. J. 14, 1682–1694. doi: 10.1111/pbi.12529, PMID: 26868756 PMC5067671

[31] Liu C. Niu G. Li X. Zhang H. Chen H. Hou D.. (2021). Comparative label-free quantitative proteomics analysis reveals the essential roles of N-glycans in salt tolerance by modulating protein abundance in Arabidopsis. Front. Plant Sci. 12:646425. doi: 10.3389/fpls.2021.646425, PMID: 34276718 PMC8283305

[32] Liu C. Niu G. Zhang H. Sun Y. Sun S. Yu F.. (2018). Trimming of N-glycans by the Golgi-localized α-1,2-Mannosidases, MNS1 and MNS2, is crucial for maintaining RSW2 protein abundance during salt stress in Arabidopsis. Mol. Plant 11, 678–690. doi: 10.1016/j.molp.2018.01.006, PMID: 29409894

[33] Mercx S. Smargiasso N. Chaumont F. De Pauw E. Boutry M. Navarre C. (2017). Inactivation of the β(1,2)-xylosyltransferase and the α(1,3)-fucosyltransferase genes in *Nicotiana tabacum* BY-2 cells by a multiplex CRISPR/Cas9 strategy results in glycoproteins without plant-specific Glycans. Front. Plant Sci. 8:403. doi: 10.3389/fpls.2017.00403 28396675 PMC5366340

[34] Metzler M. Gertz A. Sarkar M. Schachter H. Schrader J. W. Marth J. D. (1994). Complex asparagine-linked oligosaccharides are required for morphogenic events during post-implantation development. EMBO J. 13, 2056–2065. doi: 10.1002/j.1460-2075.1994.tb06480.x, PMID: 8187759 PMC395055

[35] Mølhøj M. Ulvskov P. Dal Degan F. (2001). Characterization of a functional soluble form of a Brassica napus membrane-anchored endo-1,4-beta-glucanase heterologously expressed in *Pichia pastoris*. Plant Physiol. 127, 674–684. doi: 10.1104/pp.010269, PMID: 11598241 PMC125102

[36] Nagashima Y. Ma Z. Liu X. Qian X. Zhang X. von Schaewen A.. (2020). Multiple quality control mechanisms in the ER and TGN determine subcellular dynamics and salt-stress tolerance function of KORRIGAN1. Plant Cell 32, 470–485. doi: 10.1105/tpc.19.00714, PMID: 31852774 PMC7008481

[37] Nekrasov V. Li J. Batoux M. Roux M. Chu Z. H. Lacombe S.. (2009). Control of the pattern-recognition receptor EFR by an ER protein complex in plant immunity. EMBO J. 28, 3428–3438. doi: 10.1038/emboj.2009.262, PMID: 19763086 PMC2776097

[38] Pedersen C. T. Loke I. Lorentzen A. Wolf S. Kamble M. Kristensen S. K.. (2017). N-glycan maturation mutants in *Lotus japonicus* for basic and applied glycoprotein research. Plant J. 91, 394–407. doi: 10.1111/tpj.13570, PMID: 28407380

[39] Rips S. Bentley N. Jeong I. S. Welch J. L. Von Schaewen A. Koiwa H. (2014). Multiple N-glycans cooperate in the subcellular targeting and functioning of Arabidopsis KORRIGAN1. Plant Cell 26, 3792–3808. doi: 10.1105/tpc.114.129718, PMID: 25238750 PMC4213159

[40] Saijo Y. Tintor N. Lu X. Rauf P. Pajerowska-Mukhtar K. Häweker H.. (2009). Receptor quality control in the endoplasmic reticulum for plant innate immunity. EMBO J. 28, 3439–3449. doi: 10.1038/emboj.2009.263, PMID: 19763087 PMC2776098

[41] Schoberer J. Strasser R. (2018). Plant glyco-biotechnology. Semin. Cell Dev. Biol. 80, 133–141. doi: 10.1016/j.semcdb.2017.07.005, PMID: 28688929

[42] Sim J. S. Kesawat M. S. Kumar M. Kim S. Y. Mani V. Subramanian P.. (2018). Lack of the α1,3-Fucosyltransferase gene (*Osfuct*) affects anther development and pollen viability in Rice. Int. J. Mol. Sci. 19:1225. doi: 10.3390/ijms19041225, PMID: 29670011 PMC5979348

[43] Stefanowicz K. Lannoo N. Zhao Y. Eggermont L. Van Hove J. Al Atalah B.. (2016). Glycan-binding F-box protein from *Arabidopsis thaliana* protects plants from *Pseudomonas syringae* infection. BMC Plant Biol. 16:213. doi: 10.1186/s12870-016-0905-2, PMID: 27716048 PMC5050601

[44] Stenitzer D. Mócsai R. Zechmeister H. Reski R. Decker E. L. Altmann F. (2022). O-methylated N-glycans distinguish mosses from vascular plants. Biomolecules 12:136. doi: 10.3390/biom12010136, PMID: 35053284 PMC8773788

[45] Strasser R. (2016). Plant protein glycosylation. Glycobiology 26, 926–939. doi: 10.1093/glycob/cww023, PMID: 26911286 PMC5045529

[46] Strasser R. (2018). Protein quality control in the endoplasmic reticulum of plants. Annu. Rev. Plant Biol. 69, 147–172. doi: 10.1146/annurev-arplant-042817-040331, PMID: 29570364 PMC7039705

[47] Strasser R. Altmann F. Mach L. Glössl J. Steinkellner H. (2004). Generation of *Arabidopsis thaliana* plants with complex N-glycans lacking beta1,2-linked xylose and core alpha1,3-linked fucose. FEBS Lett. 561, 132–136. doi: 10.1016/S0014-5793(04)00150-4, PMID: 15013764

[48] Strasser R. Bondili J. Schoberer J. Svoboda B. Liebminger E. Glössl J.. (2007b). Enzymatic properties and subcellular localization of Arabidopsis beta-N-acetylhexosaminidases. Plant Physiol. 145, 5–16. doi: 10.1104/pp.107.101162, PMID: 17644627 PMC1976588

[49] Strasser R. Bondili J. S. Vavra U. Schoberer J. Svoboda B. Glössl J.. (2007a). A unique beta1,3-galactosyltransferase is indispensable for the biosynthesis of N-glycans containing Lewis a structures in *Arabidopsis thaliana*. Plant Cell 19, 2278–2292. doi: 10.1105/tpc.107.052985, PMID: 17630273 PMC1955701

[50] Strasser R. Schoberer J. Jin C. Glössl J. Mach L. Steinkellner H. (2006). Molecular cloning and characterization of *Arabidopsis thaliana* Golgi alpha-mannosidase II, a key enzyme in the formation of complex N-glycans in plants. Plant J. 45, 789–803. doi: 10.1111/j.1365-313X.2005.02648.x, PMID: 16460512

[51] Strasser R. Seifert G. Doblin M. S. Johnson K. L. Ruprecht C. Pfrengle F.. (2021). Cracking the "sugar code": A snapshot of N- and O-glycosylation pathways and functions in plants cells. Front. Plant Sci. 12:640919. doi: 10.3389/fpls.2021.640919, PMID: 33679857 PMC7933510

[52] Strasser R. Stadlmann J. Schähs M. Stiegler G. Quendler H. Mach L.. (2008). Generation of glyco-engineered *Nicotiana benthamiana* for the production of monoclonal antibodies with a homogeneous human-like N-glycan structure. Plant Biotechnol. J. 6, 392–402. doi: 10.1111/j.1467-7652.2008.00330.x, PMID: 18346095

[53] Strasser R. Steinkellner H. Borén M. Altmann F. Mach L. Glössl J.. (1999). Molecular cloning of cDNA encoding N-acetylglucosaminyltransferase II from *Arabidopsis thaliana*. Glycoconj J 16, 787–791. doi: 10.1023/a:1007127815012 11229321

[54] Sun Z. Brodsky J. L. (2019). Protein quality control in the secretory pathway. J. Cell Biol. 218, 3171–3187. doi: 10.1083/jcb.201906047, PMID: 31537714 PMC6781448

[55] Takano S. Matsuda S. Funabiki A. Furukawa J. Yamauchi T. Tokuji Y.. (2015). The rice RCN11 gene encodes β1,2-xylosyltransferase and is required for plant responses to abiotic stresses and phytohormones. Plant Sci. 236, 75–88. doi: 10.1016/j.plantsci.2015.03.022, PMID: 26025522

[56] Veit C. König J. Altmann F. Strasser R. (2018). Processing of the terminal alpha-1,2-linked mannose residues from oligomannosidic. Front. Plant Sci. 9:1807. doi: 10.3389/fpls.2018.01807, PMID: 30574158 PMC6291467

[57] Viëtor R. Loutelier-Bourhis C. Fitchette A. Margerie P. Gonneau M. Faye L.. (2003). Protein N-glycosylation is similar in the moss *Physcomitrella patens* and in higher plants. Planta 218, 269–275. doi: 10.1007/s00425-003-1099-z, PMID: 14566560

[58] von Schaewen A. Sturm A. O'neill J. Chrispeels M. (1993). Isolation of a mutant Arabidopsis plant that lacks N-acetyl glucosaminyl transferase I and is unable to synthesize Golgi-modified complex N-linked glycans. Plant Physiol. 102, 1109–1118. doi: 10.1104/pp.102.4.1109, PMID: 8278542 PMC158895

[59] Wenderoth I. von Schaewen A. (2000). Isolation and characterization of plant N-acetyl glucosaminyltransferase I (GntI) cDNA sequences. Functional analyses in the Arabidopsis *cgl* mutant and in antisense plants. Plant Physiol. 123, 1097–1108. doi: 10.1104/pp.123.3.1097, PMID: 10889259 PMC59073

[60] Wilson I. Zeleny R. Kolarich D. Staudacher E. Stroop C. Kamerling J.. (2001). Analysis of Asn-linked glycans from vegetable foodstuffs: widespread occurrence of Lewis a, core alpha1,3-linked fucose and xylose substitutions. Glycobiology 11, 261–274. doi: 10.1093/glycob/11.4.261, PMID: 11358875

[61] Yoo J. Y. Ko K. S. Vu B. N. Lee Y. E. Yoon S. H. Pham T. T.. (2021). N-acetylglucosaminyltransferase II is involved in plant growth and development under stress conditions. Front. Plant Sci. 12:761064. doi: 10.3389/fpls.2021.761064, PMID: 34804097 PMC8596550

[62] Zeng W. Ford K. L. Bacic A. Heazlewood J. L. (2018). N-linked glycan micro-heterogeneity in glycoproteins of Arabidopsis. Mol. Cell. Proteomics 17, 413–421. doi: 10.1074/mcp.RA117.000165, PMID: 29237727 PMC5836367

[63] Zhang J. Wu J. Liu L. Li J. (2021). The crucial role of demannosylating asparagine-linked glycans in ERADicating misfolded glycoproteins in the endoplasmic reticulum. Front. Plant Sci. 11:625033. doi: 10.3389/fpls.2020.625033 33510762 PMC7835635

